# The evolution of intracranial aneurysm treatment techniques and future directions

**DOI:** 10.1007/s10143-021-01543-z

**Published:** 2021-04-23

**Authors:** Keng Siang Lee, John J. Y. Zhang, Vincent Nguyen, Julian Han, Jeremiah N. Johnson, Ramez Kirollos, Mario Teo

**Affiliations:** 1 Bristol Medical School, Faculty of Health Sciences, University of Bristol, Bristol, UK; 2 Department of Neurosurgery, Bristol Institute of Clinical Neuroscience, Southmead Hospital, Bristol, UK; 3 Yong Loo Lin School of Medicine, National University of Singapore, Singapore, Singapore; 4 Department of Neurosurgery, University of Tennessee Health Sciences Center, Memphis, TN USA; 5 Department of Neurosurgery, National Neuroscience Institute, Singapore, Singapore; 6 Department of Neurosurgery, Baylor College of Medicine, Houston, TX USA; 7 Division of Neurosurgery, Department of Clinical Neurosciences, University of Cambridge and Addenbrooke’s Hospital, Cambridge, UK

**Keywords:** Subarachnoid hemorrhage, Aneurysms, Clipping, Endovascular embolization, Coiling, Stents, Flow diversion

## Abstract

Treatment techniques and management guidelines for intracranial aneurysms (IAs) have been continually developing and this rapid development has altered treatment decision-making for clinicians. IAs are treated in one of two ways: surgical treatments such as microsurgical clipping with or without bypass techniques, and endovascular methods such as coiling, balloon- or stent-assisted coiling, or intravascular flow diversion and intrasaccular flow disruption. In certain cases, a single approach may be inadequate in completely resolving the IA and successful treatment requires a combination of microsurgical and endovascular techniques, such as in complex aneurysms. The treatment option should be considered based on factors such as age; past medical history; comorbidities; patient preference; aneurysm characteristics such as location, morphology, and size; and finally the operator’s experience. The purpose of this review is to provide practicing neurosurgeons with a summary of the techniques available, and to aid decision-making by highlighting ideal or less ideal cases for a given technique. Next, we illustrate the evolution of techniques to overcome the shortfalls of preceding techniques. At the outset, we emphasize that this decision-making process is dynamic and will be directed by current best scientific evidence, and future technological advances.

## Introduction

Although initially thought to occur in 1–2% of the population [13], it now appears that the prevalence of intracranial aneurysms (IAs) may range between 5 and 8% [57, 61, 127], and up to 11% [19]. IAs are the leading cause of hemorrhagic stroke, accounting for 70–85% of non-traumatic subarachnoid hemorrhages (SAH) [125]. The main treatment principle includes achieving complete IA occlusion while preserving blood flow in the parent, branching, and perforating vessels. The first surgical treatment of an IA was performed in 1933 by Norman Dott [31], who wrapped a ruptured IA, while the first obliterative clipping of an IA was performed in 1938 by Walter Dandy [26]. In 1975, Yasargil and Fox described the classic microscopic assisted open approaches for IA clipping, such as the pterional craniotomy (PTC), which afforded safe and effective exposure of the circle of Willis through the Sylvian fissure with minimal retraction on the frontal and temporal lobes [134]. Indeed, with technological developments in vascular imaging, intraoperative navigation, and fluorescence angiography, this technique remains popular for treating IAs of the anterior circulation [77, 133].

However, the more recent advance in IA treatment has been the advent of safe and effective endovascular techniques for the treatment of IAs, particularly of the posterior circulation. Acceptance and popularity of endovascular techniques increased in 2002 with the results of the International Subarachnoid Aneurysm Trial (ISAT) [73]. Although both surgical and endovascular IA occlusion technologies are effective for appropriately selected patients, improved technology for endovascular devices and techniques has shifted the balance towards these less invasive techniques with the benefit of reduced operative time, patient preference and tolerability in less healthy patients, and decreased length of in-hospital stay [73, 93, 109–111, 113]. Nonetheless, open surgical techniques including complex IA treatment with bypass techniques remain vital in the cerebrovascular neurosurgeon’s armamentarium for IAs that cannot be treated by endovascular means. The treatment techniques and management guidelines for IAs have thus continued to evolve in recent years, and these rapid developments have forced clinicians to adapt their surgical decision-making.

The purpose of this review is to provide practicing neurosurgeons with a summary of the techniques available, and to aid decision-making by highlighting ideal or less ideal cases for a given technique. Next, we illustrate the evolution of techniques to overcome the shortfalls of preceding techniques. At the outset, we emphasize that this decision-making process is dynamic and will be directed by current best scientific evidence, and future technological advances.

## Treatment strategies for IAs

Treatment options for IAs are either surgical or endovascular, and both techniques have undergone major development in recent years, which are summarized in the following sections.

## Transluminal embolization techniques

A summary of the current state of the endovascular techniques, their indications, evolution, and current limitations, is presented in Table 1.

**Table 1:** Summary of the current state of endovascular techniques, their evolution, and current limitations

Technique	Indications	Efficacy	Morbidity	Limitations	Evolution
Coiling	Saccular aneurysms Possibly regarded as “first call” treatment of intracranial aneurysms Favorable factors include: Patient-specific: • Older patients (> 70 years) Aneurysm-specific factors: • Endovascularly accessible and with favorable dome/neck ratio and aspect ratios • Posterior circulation • No space occupying hemorrhage	Coiling is a more suitable treatment for unruptured IAs in terms of complications and morbidity, especially in the short term • 70% complete occlusion rates • 30% recurrence rate • 10% retreatment rate • Complete occlusion and recurrent rates are improved with BAC (80% and 15%) • Complete occlusion rate from SAC is 60% only, but it affords lower recurrence rate (10%) • Similar rates for double catheter technique	• Simple coiling has lower risks of morbidity than open surgical clipping (0.2% mortality; 2% technique-related morbidity/complications; 15% thrombosis rates) • BAC has similar rates of complications and morbidity as simple coiling • SAC requires use of antiplatelet therapy and consequently has greater periprocedural rate of hemorrhagic complications (30%), added thrombosis complications (30%), and 2% mortality	Unfavorable factors include: Aneurysm-specific factors: • Size: too small, too large (giant) • Partially thrombosed • Wide-neck aneurysms including bifurcation IAs • Thin and fragile walls of blister IAs • Lack of true neck in dissecting and fusiform IAs Other factors: • SAC requires dual antiplatelet therapy with its inherent hemorrhagic complications • Need for repeated follow-up and coiling due to coil compaction leading to aneurysm recanalization and regrowth • Cost issues	BAC/SAC and double microcatheter technique, to overcome the IA configurations such as: • Giant IAs • Wide neck • Presence of vital vessels branching from the fundus
Flow diverters	Favorable factors Aneurysm-specific factors: • Not amenable to simple coiling or BAC/SAC • Giant IAs, fusiform IAs, and possibly bifurcation IAs • Cavernous and ophthalmic region • Wide neck • Dissecting	• 85–95% complete occlusion (FD alone) • 75–90% complete occlusion (FD + coils) • 80% favorable clinical outcome (GOS 4–5/mRS 0–2)	• 5% thrombosis • Delayed aneurysm rupture is a complication associated with IAs treated with flow-diverting stents (5% for FD alone; 3% if adjunctive with coils) • 5% morbidity	Unfavorable factors include: Aneurysm-specific factors: • Posterior circulation IAs due to numerous perforators • Bifurcation IAs • Distal IAs Technical factors: • Significant tortuosity causing suboptimal deployment and technical failure • Long learning curve Other factors: • High costs of PED and SFD • Need for long-term dual antiplatelet therapy	Some evolutionary changes: 1. To prevent thromboembolic complications—Pipeline Shield Technology, Phenox Hydrophilic Polymer Coating 2. Distal IAs—Smaller FDs such as SILK Vista Baby, FRED Jr, Phenox p48 3. Resheathable—Pipeline Flex, SILK, FRED, Phenox p64 4. Reduced porosity—SILK, Surpass Streamline
Intrasaccular flow disruptors	Aneurysm-specific factors: • Not amenable to simple coiling or BAC/SAC • Primarily for wide-necked IAs, bifurcation IAs, and sidewall IAs	• 80% occlusion rates • 10% recurrence rate • 10% retreatment rate There is little risk posed to surrounding perforators, and antiplatelet medication is not required after the procedure	1% WEB-related morbidity (unruptured) 0% WEB-related mortality (unruptured) 3% WEB-related morbidity (ruptured) 1% WEB-related mortality (ruptured) • 30% rate of WEB-related complications such as device protrusion, sac perforation, and thromboembolism	Unfavorable factors include: Aneurysm-specific factors: • Tortuous anatomy, e.g., ACommA aneurysms, as difficult to navigate with the large microcatheters	Common device is the WEB. Another intrasaccular flow disruptor is the Luna AES

*ACommA*, anterior communicating artery; *AES*, aneurysm embolization system; *BAC*, balloon-assisted coiling; *CAMEO trial*, Cerebral Aneurysm Multicenter European Onyx trial; *PED*, pipeline embolization device; *SAC*, stent-assisted coiling; *SFD*, silk flow diverters; *WEB*, Woven Endoluminal Bridge

### Simple coiling

In 1990, a detachable bare platinum coil device (Guglielmi) was introduced into clinical practice. Since then, endovascular treatment with coils has gained worldwide acceptance as an effective treatment for IAs [10, 11, 73], and coil variations have been introduced into the market which include polymer-coated coils that incite tissue response across the IA neck, and coils coated with a hydrophilic gel that expands on exposure to blood, reducing dead space.

**Fig. 1:**
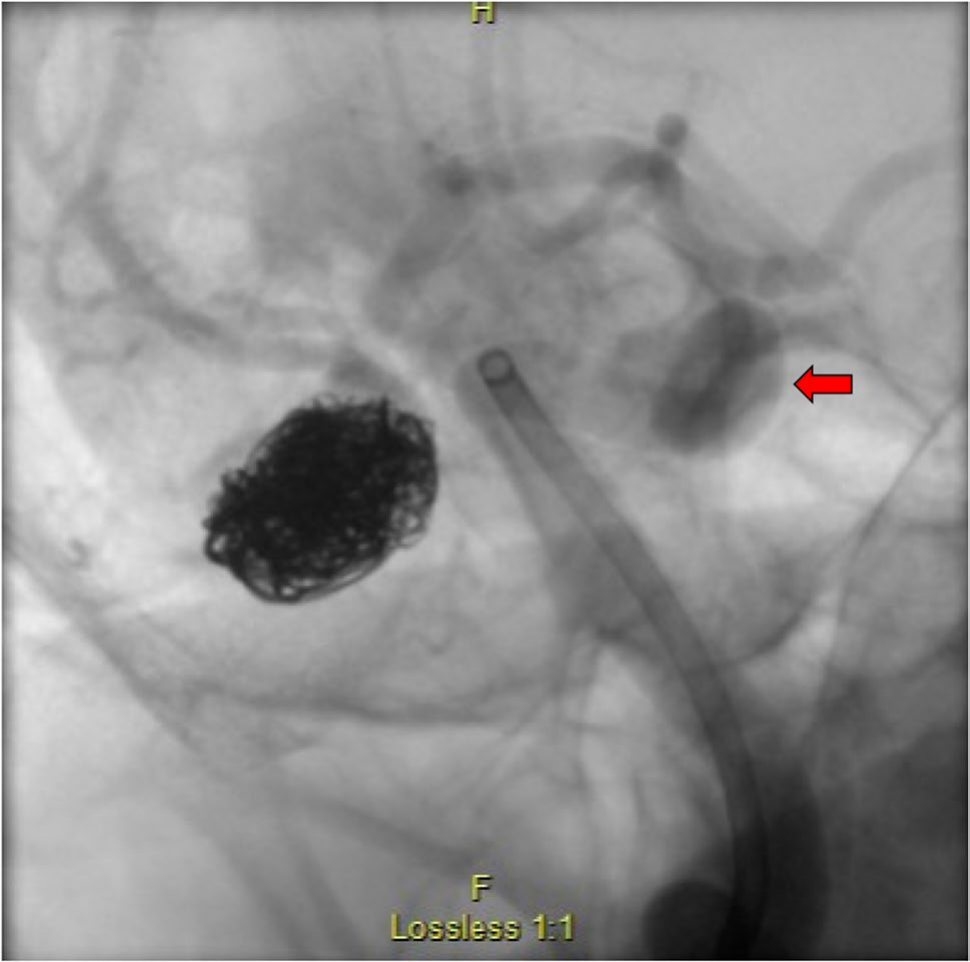
A large right ruptured MCA aneurysm was treated with a total of 19 coils. The red arrow points to an incidental right paraclinoid unruptured aneurysm

The goal in coiling is to achieve dense packing through the delivery of detachable platinum wires, resulting in an unorganized thrombus and granulation tissue formation, to limit blood circulation to the IA lumen. IAs are protected from rupture in ruptured [15, 73] and in some unruptured cases [37, 44, 132]. Clinically, packing density is recommended to be 20% or more of the IA’s volume, which can require deployment of multiple coils depending on the size of the IA (Fig. 1) [78]. Online calculators are also readily available to estimate IA volumes and packing densities for various coils and IA morphologies that can help (e.g., angiocalc.com) [4].

Coiling is increasingly popular but does come with several shortcomings. Not all IAs are completely cured at first treatment necessitating post-treatment surveillance imaging and, in a minority, may require retreatment [73, 120]. Routine follow-up imaging of a treated IA is usually performed noninvasively by either CTA or MRA. There exist no guideline and no scientific data defining the optimal regime how frequently and how long to follow-up, but generally, the frequency is dictated by the completeness and method of the original repair, as well as the other patient and IA-specific factors [107]. Another drawback of coiling is the need for retreatment due to coil compaction or IA recurrence, hence also further necessitating follow-up [15, 22, 40, 73, 95, 106, 109]. A second treatment of IAs has been reported to be around 12%, on average of 27 months after the previous procedure [40]. Hence, the typical scheme would include a first follow-up scheduled 3–6 months after the endovascular procedure, followed by a 12–24-month follow-up and a midterm follow-up at 3–5 years [56]. The combination of invasiveness and radiation make digital subtraction angiography (DSA) less readily used for follow-up, but is useful to ascertain an accurate estimation of the size of the IA remnant. If not diligently followed post-treatment, ruptured IA patients treated with coiling may be at risk of delayed IA recanalization and recurrent SAH [73].

Possible determinants for initial incomplete IA occlusion and complications are unfavorable IA anatomy and vessel geometry as well as the types of coils that are used. Possible risk factors for recurrence of a coiled IA over time are large IA size [95, 123], presence of intraluminal thrombus, low packing density [49], initial incomplete occlusion [38], duration of follow-up [89], ruptured IAs, location in the posterior circulation [15], and a large neck–dome ratio [33, 47, 96].

There are several major complications associated with coil embolization: thromboembolism, perforation of the IA, early rebleeding, parent artery obstruction, collapsed coils, coil malposition, even coil migration.

### Balloon-assisted coiling

**Fig. 2:**
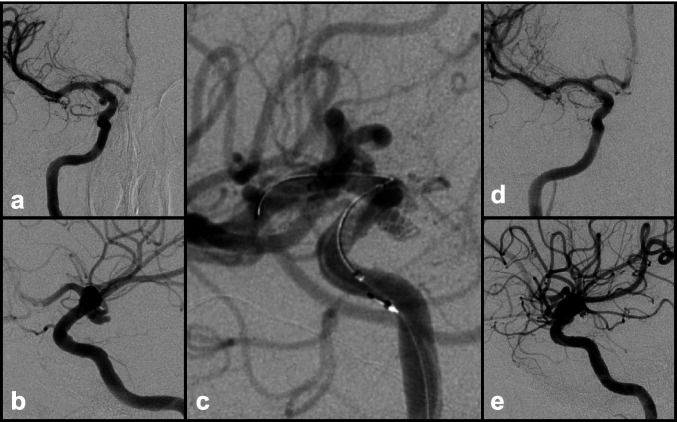
Ruptured, wide-neck 4-mm right PCommA aneurysm in a poor surgical candidate. **a** AP and **b** lateral views. The initial framing coil would not stay in the aneurysm; thus, a balloon was inflated during placement of the initial framing coil allowing it to **c** remain within the aneurysm. **d** AP and **e** lateral views after subsequent coils were placed obliterating the dome. Dome remains obliterated at 2-year follow-up

Balloon-assisted coiling (BAC) or balloon remodeling is a method originally described in the cardiac literature and subsequently adapted for use in the cerebrovascular field for the treatment of IAs with a wide neck, when popularized by Moret et al. [74]. During placement of the coil, a compliant balloon is inflated in the parent vessel lumen to create a temporary IA neck allowing the coil to frame inside the IA and preventing it from herniating out into the parent vessel. This technique is particularly useful when treating ruptured IAs with unfavorable anatomy for stand-alone coiling. Several special types of balloons such as hypercompliant, round-shaped, and double lumen balloons are used depending on the situation (Fig. 2) [142].

Although BAC has increased the capacity to coil IAs previously considered to be unfavorable for endovascular treatment, some studies have suggested higher complication rates in BAC compared to simple coiling. BAC is associated with an increased risk of long-term coil compaction and recanalization due to limited filling of the IA volume [23]. The Analysis of Treatment by Endovascular approach of Non-ruptured Aneurysms (ATENA) study showed a higher intraoperative aneurysmal IA rupture rate with BAC than with simple coiling [85]. However, this difference was not statistically significant, nor was there a significant difference in morbidity and mortality rates between the groups. However, more recent large analyses have demonstrated BAC to be safe and effective in treating IAs with an unfavorable dome-to-neck ratio [17, 129, 130].

### Stent-assisted coiling

**Fig. 3:**
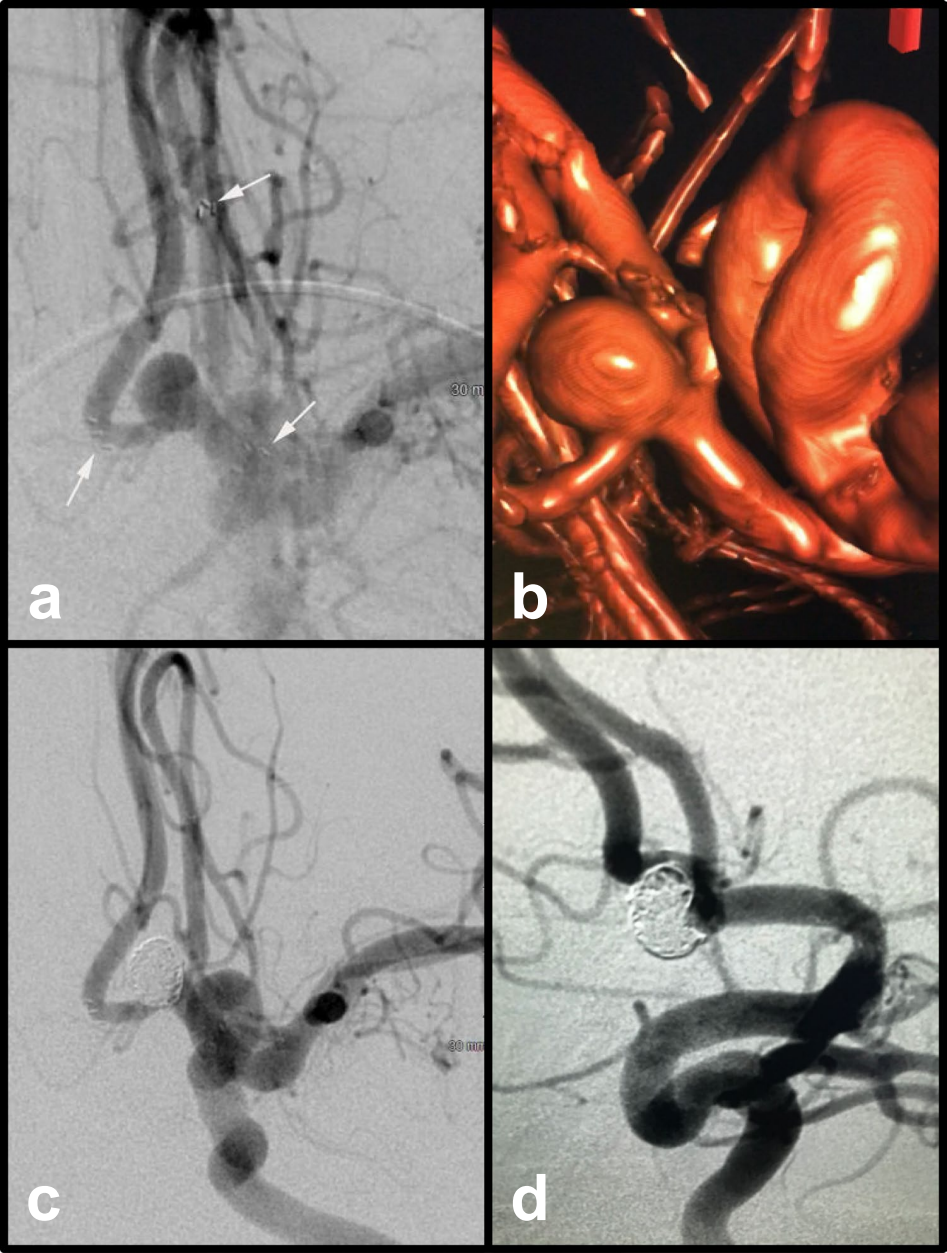
Unruptured 7-mm, wide-necked ACommA aneurysm. **a** AP view demonstrating Y configuration stents with distal markers visible in each A2 segment (arrows) and proximal maker in the A1 (arrow) and **b** a 3D view of the neck. Post-coiling **c** AP and **d** lateral view demonstrating aneurysm obliteration without encroachment of the parent vessels

The first coronary stent was implanted in a human in 1986, and by the twenty-first century, its use had increased to over 80% of all endovascular interventions in coronary arteries [42]. The first report of stent-assisted coiling (SAC) used for IAs was published by Higashida et al. only in 1997, for a ruptured fusiform IA of the basilar artery [41]. However, it was only in late 2002 that the first stent was actually designed for treatment of IAs. This was the Neuroform stent, which received a Humanitarian Device Exemption from the Food and Drug Administration (FDA). Stents in association with coils have been used to treat wide-neck IAs that cannot be treated by coils alone or with BAC. Stent cell design can be divided into open-cell and closed-cell types which have different physical properties. Closed-cell stents are characterized by small free cell areas between the struts, whereas open-cell stents have larger uncovered gaps. The primary objective of a stent application is to provide a scaffold to protect the parent vessel and permit coiling of wide-necked, giant, fusiform, and some other complex IAs, which are not amenable to coiling alone (Fig. 3). There are various configurations to achieve this (R-stent, L-stent, nonoverlapping-Y, virtual-Y, horizontal, kissing-Y which is a double stent placement in a parallel fashion, crossing-Y (R to L), and crossing-Y (L to R) models). The principal limitation of simple coiling is the high IA recurrence, and hence, the adjunct use of stents for coil aims to reduce this recurrence [83]. More recently, novel endovascular devices have also been approved to specifically treat bifurcation IAs with wide necks. Among these devices, the pCONus1 and pCONus2 are stent-like self-expanding nitinol implant with four to six distal petals allowing coiling of the aneurysmal sac [108]. The PulseRider is another self-expanding nitinol implant with frame configuration that opens to conform to the vessel walls. These devices were specifically engineered to preserve luminal patency and hemodynamic flow through the parent vessel bifurcation, while minimizing exposed metal to expedite endothelialization [36, 91]. Exciting new devices include the pCANvas, pCONUSTM, eClipsTM, and Comaneci. While larger institutional data are required, recent meta-analyses have demonstrated high rates of technical success and sufficiently low rates of morbidity and mortality with their use in wide-neck IAs [91, 108].

In a meta-analysis comparing the outcomes of 2698 SAC and 29,388 unassisted coiling patients [83], Phan et al. reported that both SAC and unassisted coiling were comparable in terms of similar immediate occlusion and complication rates. There were significantly lower rates of IA recurrence in SAC compared to coiling only (OR = 0.43; 95% CI: 0.28–0.66). However, progressive thrombosis was significantly more likely in SAC (29.9%) compared to unassisted coiling (17.5%) (OR = 2.71; 95% CI: 1.95–3.75). SAC is also associated with a higher mortality rate (OR = 2.16; 95% CI: 1.33–3.52).

However, while these devices provide another treatment option for endovascular repair, additional risks are associated with stent placement compared to coiling alone. Despite the generations of intracranial stents which have evolved over time, there are still a number of limitations with the current stent devices with rare complications, including stent migration, vessel trauma, thrombosis, and in-stent restenosis having been reported [8, 18, 35, 71].

With the use of stents or flow diverters, complication rates tend to be higher than with simple coiling or BAC due to metallic scaffold present in the parent vessel. The inherent thrombogenicity of these devices necessitates use of dual antiplatelet medication perioperatively and post-procedure to prevent thromboembolic events. The need for antiplatelet therapy limits the role of stent placement in patients with ruptured IAs as it increases the risk of the procedure as well as the need for adjunctive therapies such as external ventricular drains or CSF diversionary shunts [58, 136]. Newer stents with modified surfaces to reduce their thrombogenicity can potentially be safely used with only a single antiplatelet agent. This application could potentially overcome some of the existing challenges when using flow diverters in the treatment of ruptured aneurysms [70].

### Double microcatheter technique

The double microcatheter technique is an alternative to treat IAs with complex configurations, not amenable to simple coiling, such as a wide neck or the presence of vital vessels branching from the fundus [6, 52, 104]. This technique involves creating a stable coil frame using two coils to brace each other [135]. Prior to coiling, two microcatheters are placed within the proximal and distal aspects of the IA dome. The first coil is positioned proximally to form a supporting frame, while the other coils are deployed through the distal microcatheter. The coil frame is kept in place until packing is satisfactorily completed [142].

Besides its use for IAs with unfavorable configurations, a recent study of 85 patients demonstrated efficacy of the double microcatheter technique in treating very small IAs of ≤ 3 mm [135]. This technique has also been shown to be safe and effective in the treatment of a case of elongated middle cerebral artery (MCA) bifurcation IA [54]. However, care should be taken to prevent shifting of coils when disengaging the distal microcatheter. Additionally, concerns have also been raised about decreased coil packing density and increased recurrence rate using this technique.

### Flow diverters

**Fig. 4:**
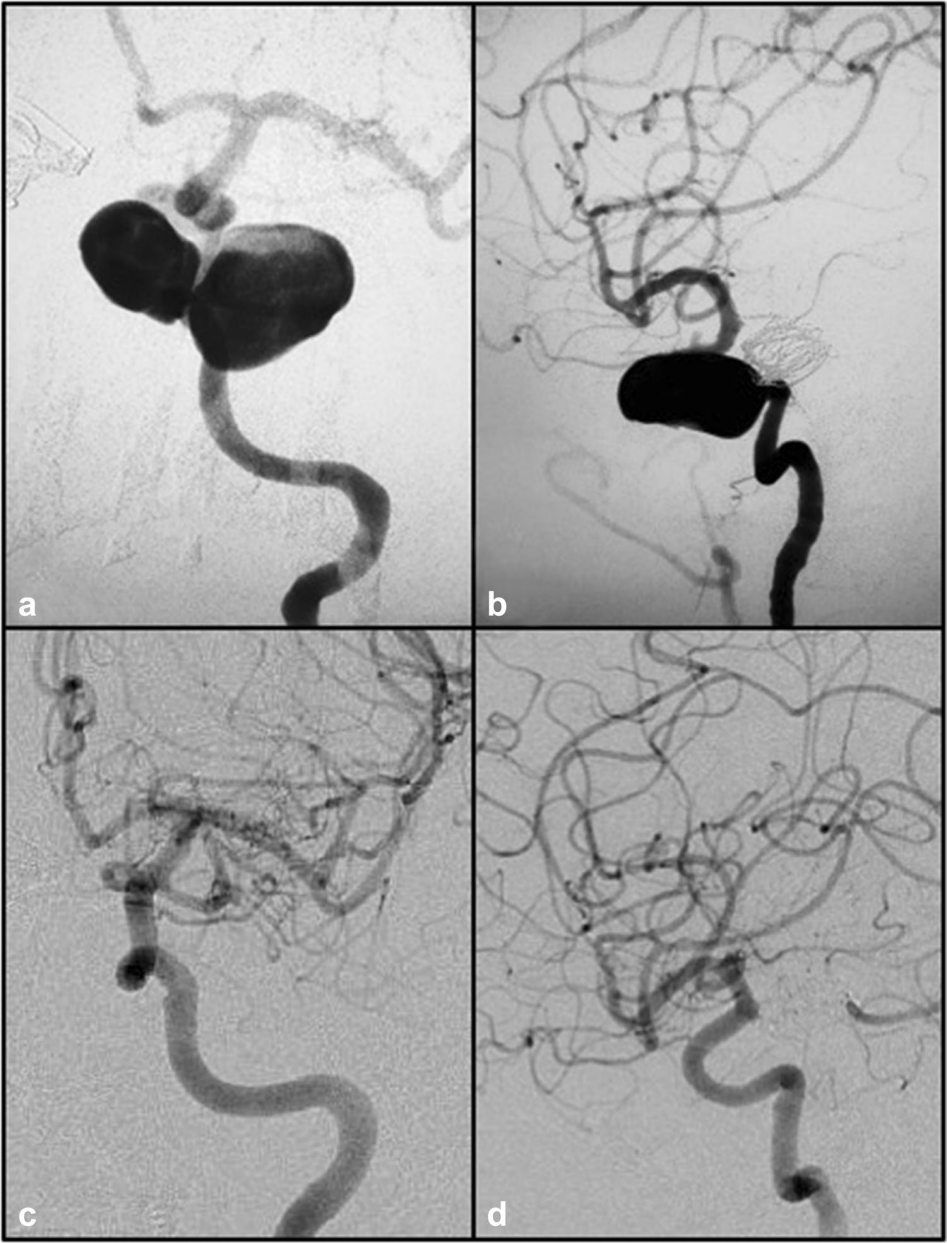
**a** AP and **b** lateral pre-treatment angiogram demonstrating a giant left cavernous segment ICA aneurysm. **c** AP and **d** lateral projections of the left ICA 6 months after flow diversion treatment demonstrating resolution of the aneurysm and remodeling of the parent vessel

With the advent of computational fluid dynamics and improvement in imaging modalities over the past few decades, more research has begun focusing on medical device design. Flow diverters are a new generation of neuroendovascular devices that consist of highly flexible tubular structures with mesh and are very similar to stents in terms of engineering design [139]. A major difference is that the mesh is less porous than in typical stents. Therefore, the major focus in manufacturing design for flow diverters has been shifted from solid mechanics to fluid mechanics (Fig. 4).

The primary goal of a flow diverter is to shunt flow away from the IA by placing a mesh structure, similar to a stent, on the IA neck along the parent artery. By decoupling blood flow between the parent artery and aneurysmal sac, a flow diverter can create blood stasis in the IA to allow for thrombus formation inside the IA. The IA gradually thromboses and regresses over time, while the device acts as a scaffold for endothelialization across the IA neck resulting in parent vessel remodeling and IA resolution. Flow diverters are intended to be used in anatomical situations where SAC becomes difficult, and hence are suitable for use in wide-necked, giant, or fusiform IAs. Symptomatic cavernous segment IAs are preferentially treated with this technique, and ophthalmic region IAs are increasingly being treated by flow diversion rather than open surgical approaches. Additionally, dysplastic or fusiform IAs in other locations not amenable to straightforward endovascular or open vascular treatment are increasingly being treated in this manner.

In a recent meta-analysis, Madaelil et al. reported the use of flow diverters for 126 distinct ruptured IA cases, including dissecting (28%, 35/125), fusiform (10%, 12/125), giant (3%, 4/125), blister (38%, 47/125), and saccular (22%, 27/125) types [69]. IA rerupture occurred in 5% of the IAs, with 67% of these reruptures occurring in IAs measuring > 2 cm. With a median follow-up of 6 months, complete occlusion of the IA was achieved in 90% of patients on follow-up clinical imaging, and favorable clinical outcome was attained in 81% of patients. Another meta-analysis assessing the outcomes of early (within 2 days) versus delayed flow diversion for ruptured IAs showed no statistical difference in overall hemorrhagic complication and stroke rates [30].

As mentioned, flow diverters are typically deployed in situations where established techniques are not viable options. It is not surprising that, with increased technical demands, deployment of flow diverters also comes with increased technical complications. Yet a recent meta-analysis still established its effectiveness and low associated morbidity/mortality risks, in the use of US commercially available flow diverters predominantly in unruptured small/medium ICA IAs. At 12 months, occlusion was achieved in 74.6% (95% CI 66.8 to 81.7%) of cases with low rates of primary safety events, at 7.8% (95% CI 4.8 to 11.4%) [34]. These recent findings are reassuring that flow diverters remain a viable option.

Delayed IA rupture (DAR) has been reported to be a complication associated with IAs treated with flow-diverting stents [97]. The mechanism of DAR is still not entirely elucidated but is likely due to the higher porosity of early available devices, limiting the flow-diverting effect [25]. In vitro studies have suggested that stent porosity and local hydrodynamic conditions play an important role in uncoupling flow between the parent vessel and the aneurysmal sac [122]. Further improvements of currently available devices (PED) have created less porous devices—Surpass (Stryker), p64 (Phenox), FRED (flow redirection endoluminal device)—obviate some of these earlier issues [94].

### Intrasaccular flow disruptors

**Fig. 5:**
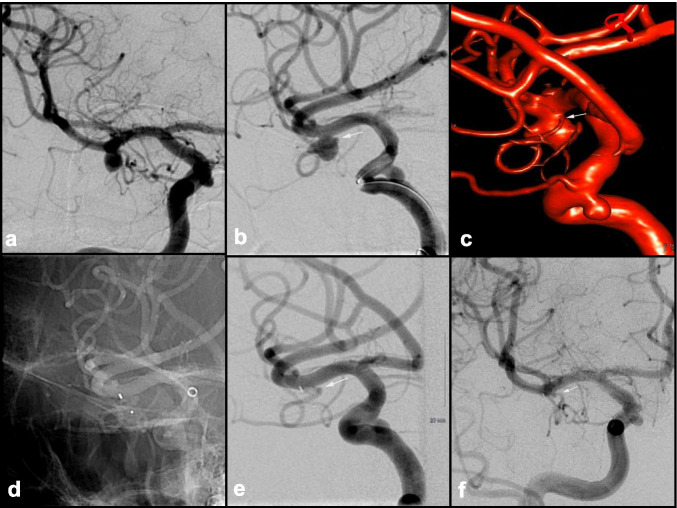
Pre-treatment **a** AP, **b** lateral, and **c** 3D images of a right anterior temporal artery. MCA aneurysm with the anterior temporal artery originating from the neck (arrow). Immediate post-WEB device deployment **d** unsubtracted image showing the device and **e** subtracted lateral and image demonstrating aneurysm obliteration with patency of the branching artery (arrow). Six-month follow-up **f** AP image showing continued occlusion with patent anterior temporal artery (arrow)

Intrasaccular flow disruption is a recently developed concept primarily proposed for the treatment of bifurcation IAs [62, 79, 86]. One such device commonly discussed is the Woven Endoluminal Bridge (WEB) (Fig. 5), which is deployed inside the aneurysmal sac to divert flow away from the dome leading to stagnation and thrombosis, and can be used for the treatment of saccular or wide-necked bifurcation IAs, and even ruptured IAs [141]. There is little risk posed to surrounding perforators, and antiplatelet medication is not required after the procedure [84]. A multicenter European study demonstrated adequate occlusion of 89.7% at midterm follow-up of median 13 months, and good clinical outcome (mRS < 2) of 93.3% at last follow-up [67]. The recent Woven EndoBridge Intrasaccular Therapy (WEB-IT) study demonstrated promising results, with only one (0.7%) primary safety event in 148 patients which was a delayed ipsilateral parenchymal hemorrhage. No further primary safety events occurred beyond 30 days through 1 year. At the 1-year angiographic follow-up, 84.6% of patients had adequate occlusion with complete occlusion in 53.8% of subjects [5]. The safety and effectiveness of the WEB are also corroborated by other studies [87, 126].

An upcoming intrasaccular flow disruptor is the Luna Aneurysm Embolization System (AES), which is a self-expanding ovoid braided implant that provides an attenuated mesh of metal across the IA neck when placed into the IA cavity. In a preliminary study, the Luna AES achieved high rates of complete angiographic occlusion in 12 rabbit IA models [53]. A more recent prospective multicenter study of 64 bifurcation and sidewall IAs demonstrated adequate occlusion of 79.2%, with a morbidity rate of 1.8% and mortality of 1.6% at 36 months [88]. Further evidence is anticipated to evaluate the efficacy and safety of this device.

## Surgical techniques

In the previous sections, we have discussed the use of BAC, SAC, and flow diversion to treat IAs with complex morphologies such as large, wide-necked, and fusiform IAs. Endovascular therapies have undoubtedly given multidisciplinary treatment teams important new treatment options. However, there remain several limitations to these methods that need to be addressed. BAC has been reported to be associated with an increased risk of long-term coil compaction and recanalization due to limited filling of the IA volume [23]. Meanwhile, SAC has been shown to have a greater periprocedural rate of hemorrhagic complications with the use of antiplatelet therapy [75]. Due to these limitations, treatment of more complex IAs such as bifurcation and sidewall IAs remains a challenge [2, 27, 100].

A summary of the current state of the microsurgical techniques, their indications, evolution, and current limitations, is presented in Table 2.

**Table 2:** Summary of the current state of microsurgical techniques, their evolution, and current limitations

Technique	Indications	Efficacy	Morbidity	Limitations	Evolution
Clipping	Wide variety of mostly saccular IAs Possible factors considered: Patient-specific: • Younger patients (< 70 years) Aneurysm-specific factors: • Saccular IAs, • Giant IAs • No key perforating vessels incorporated in sac • Space-occupying hemorrhage • Located on the MCA, and pericallosal artery	95% complete occlusion rate 5% residual necks < 1% bleeding from residual necks 1–5% retreatment rate Temporary clip occlusion of the proximal artery can minimize the risk of intraoperative aneurysm rupture and maximize visualization for aneurysm neck dissection	< 1% mortality 3–5% morbidity DCI has been shown to be more common in patients treated with clipping than endovascular approaches Temporary clip occlusion of the proximal artery poses potential risks of ischemia in the vascular territories supplied by the proximal artery	Unfavorable factors include: Patient-specific: • Older patients (> 70 years) Aneurysm-specific factors: • Posterior circulation • Fusiform IAs • Some giant IAs incorporating branches in the neck • Aneurysms with key perforating vessels	Several techniques have been developed to optimize clipping • Modification of skull base approaches and evolution of minimally invasive and endoscopic-assisted approaches • Neuroprotection + neuromonitoring and achieving intraoperative proximal control • Temporary clip ligation of the proximal artery • Temporary cross-clamping of the extracranial carotid artery in the neck • Endovascular balloon occlusion • EC-IC bypass • ICGVA with fluorescein video angiography • Intraoperative DSA (if available) could be a good intraoperative adjunct
EC-IC bypass techniques	Patient-specific: • Younger patients (< 70 years) Aneurysm-specific factors: • Giant IAs Technical factors: • Technically demanding, including deep anastomosis • Donor graft harvest usually from distant site (radial artery or saphenous vein)	Shi et al. showed that, in 93 patients who had undergone bypass for giant IAs, the patency rate was 96%	5–10% morbidity	Aneurysm-specific factors: • Located on ACA, MCA, PICA, and basilar apex Patient-specific: • Older patients (> 70 years)	There are two kinds of EC-IC bypass: low-flow and high-flow bypass Low-flow bypass involves anastomosing the STA to an intracranial artery such as the MCA High-flow bypass connects the CCA or the ECA to an intracranial artery with the use of a conduit such as the GSV or the RA
IC-IC bypass techniques	Patient-specific: • Younger patients (< 70 years) Aneurysm-specific factors: • Giant IAs • Located on ACA, MCA, PICA, and basilar apex Technical factors: • In situ donor graft, for example, adjacent pericallosal or PICA vessels	High aneurysm obliteration rates, high bypass patency rates, and good neurological outcomes. Could be more favorable in selective cases compared to EC-IC bypass	5–10% morbidity	Patient-specific: • Older patients (> 70 years) Technical factors: • Technically challenging procedure	The role of IC-IC bypass in aneurysm surgery was introduced more recently, compared to EC-IC bypass

*CCA*, common carotid artery; *DCI*, delayed cerebral ischemia; *DSA*, digital subtraction angiography; *ECA*, external carotid artery; *EC-IC*, extracranial-to-intracranial; *GSV*, great saphenous vein; *ICGVA*, indocyanine green video angiography; *IC-IC*, intracranial-to-intracranial; *MCA*, middle cerebral artery; *PICA*, posterior inferior cerebellar artery; *RA*, radial artery; *SAH*, subarachnoid hemorrhage; *STA*, superficial temporal artery

### Microsurgical clipping

**Fig. 6:**
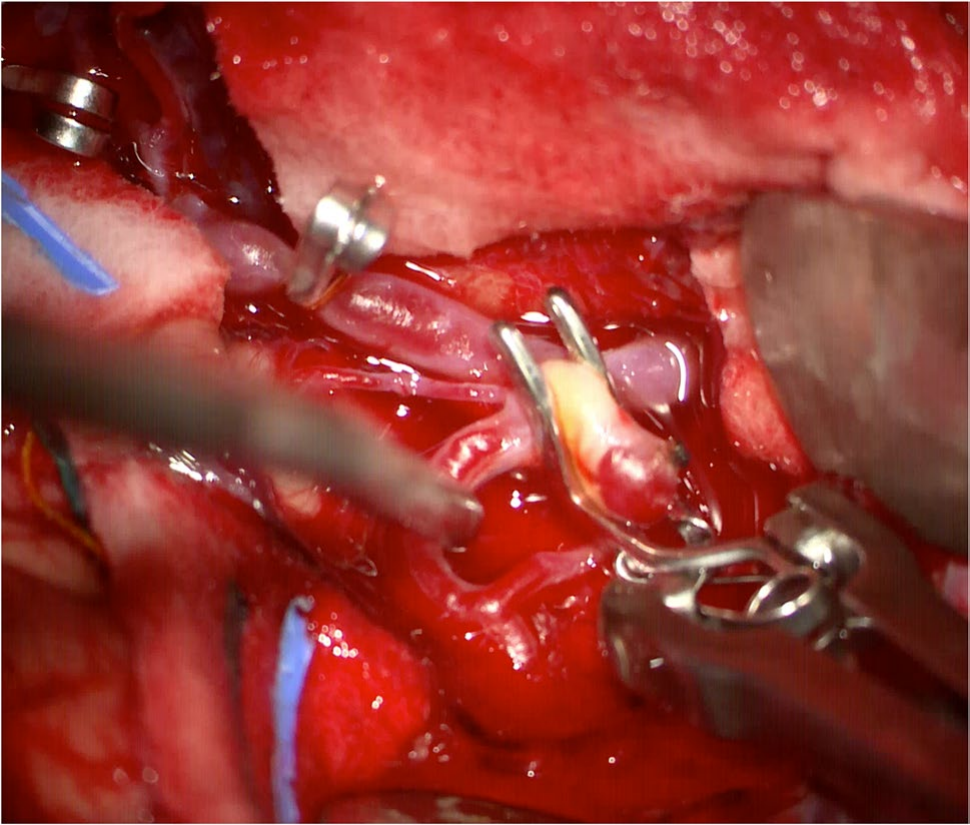
Clipping of a partially calcified MCA bifurcation aneurysm is demonstrated here with temporary clipping of the M1 trunk to soften the aneurysm dome. In the upper left corner, a permanent clip is seen on a posterior communicating artery (PCommA) aneurysm (not shown) that was concomitantly clipped during the same operation

Clipping of IAs is the most conventional and established method of IA treatment, with the first of such procedures dating back to 1937 [66]. It was not until 1975, when Yasargil and Fox introduced the microscope in the neurosurgeon’s arsenal, which afforded safe and effective exposure of the circle of Willis [134]. Principles of clipping involve gaining access to the IA via an open craniotomy, dissecting the IA away from surrounding parenchyma, optimizing visualization of the IA neck, and employing a metallic clip permanently to completely obliterate the neck without compromising the patency and integrity of the parent and perforating vessels (Fig. 6) [28].

Initial microsurgical techniques have improved with the modification of clip designs and microinstruments. There has been further evolution of access from modified skull base approaches to the so-called minimally invasive approaches to the use of endoscopy-assisted surgery [72]. This has been concomitantly associated with improvement in “neuroprotection” and novel techniques of achieving intraoperative proximal control for complex lesions. Some of these include the complementary role of endovascular techniques such as the use of intraluminal balloons for proximal control.

Several techniques have evolved as an adjunct of microsurgical clipping, to aid the surgeon in optimizing outcomes. These include temporary clip ligation of the proximal artery, temporary cross-clamping of the extracranial carotid artery in the neck, endovascular balloon occlusion with suction, and extracranial-to-intracranial bypass as well as cardiac standstill [7, 39, 50]. These will be further described below. Clipping is versatile and is a viable option for most types of IAs, including saccular IAs, giant IAs, and fusiform IAs without key perforating vessels [142]. Although reserved for fit and younger patients, microsurgical clipping is more suitable than coiling in patients with expanding hematoma or signs of raised intracranial pressure requiring urgent decompression [1, 76, 81, 116].

Temporary clip occlusion of the proximal artery is one of the most popular adjunct microsurgical clippings of IA, in order to minimize the risk of intraoperative IA rupture. By temporarily occluding the proximal vessel, blood supply to the IA is obliterated, causing the IA to soften and thereby maximizing visualization and IA neck dissection. However, this technique poses potential risks of ischemia in the vascular territories supplied by the proximal artery. To minimize the risk of ischemic complications, the occlusion time is typically kept to below 10–20 min [118]. In challenging cases where a single occlusion episode is insufficient, the use of multiple episodes of occlusion with 15-min reperfusions in between has been shown to be safe and effective [90]. With cerebral protection of hypothermia, anesthesia-induced burst suppression, combined with raising baseline blood pressure to encourage collateralization, longer temporary occlusion time is generally tolerated (in the event of microvascular anastomosis during cerebral bypass). However, the Intraoperative Hypothermia for Aneurysm Surgery Trial (IHAST) demonstrated that hypothermia aimed at neuroprotection during IA surgery did not improve the neurologic outcome after craniotomy among WFNS grade 1–3 patients with SAH [121]. Intraoperative rupture of an IA during microsurgery may have devastating consequences. Short-term cardiac arrest induced by intravenous adenosine with rapid cardiac pacing has been recently proposed as a feasible method to aid the surgeon, and has been demonstrated to be superior to temporary clipping during IA surgery [9, 43, 68].

Advances in neuromonitoring are instrumental in providing safer surgery. More recently, the development of infrared indocyanine green video angiography (ICGVA) is increasingly finding its way as a quality control addition. ICGVA is a relatively new advancement in the field of cerebrovascular surgery with its first use in 2005 for intraoperative evaluation of vascular patency [92]. In a preliminary analysis of 114 patients, Raabe et al. demonstrated the effectiveness of ICGVA in providing real-time feedback on the patency of both the parenting and perforating vessels as well as information regarding the IA sac [92]. However, since then, several studies have raised concerns against the use of ICGVA as a single modality for intraoperative evaluation during IA clipping [55, 102, 131]. In one such study, Washington et al. demonstrated an ICGVA intraoperative angiography dis-concordance rate of 14.3%, requiring post-intraoperative angiography adjustment of the IA clip [131]. These studies advocate the use of intraoperative digital subtraction angiography (DSA) as the gold standard for IA surgery, and suggest the use of fluorescein video angiography as a complementary technique to ICGVA in certain cases. A hybrid operating room might provide future solution for on-table DSA post IA clipping, though this is not current standard practice. Acknowledging the occurrence of technical mishaps during IA clipping [14], the use of ICG has allowed diminishing morbidity by modification of clip placement [117].

### Bypass techniques

Bypass techniques used in cerebrovascular neurosurgery can be extracranial-to-intracranial (EC-IC) or intracranial-to-intracranial (IC-IC). As these two types have clinically distinct indications and technical strategies, they are discussed separately.

**Fig. 7:**
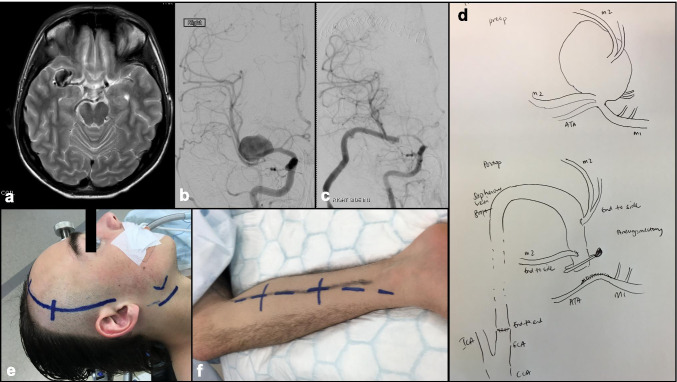
An 18-year-old male presented with intermitted left-sided weakness. **a** MRI brain showed ischemic changes within the right MCA territory with wide-necked, complex MCA aneurysm. **b** Preoperative cerebral angiogram (right ICA injection) showed complex MCA aneurysm with MCA branches arising from the body of the aneurysm. **c** Right ECA-saphenous vein-MCA bypass (EC-IC bypass) was performed, with postoperative cerebral angiogram confirmed right MCA perfusion by high-flow bypass graft. **d** Schematic diagram to illustrate the complex aneurysm configuration, and bypass strategy, implanting the MCA branches into the saphenous vein graft. **e**, **f** Intraoperative view of the cranial incision, neck incision, and right saphenous vein harvest. He made good postoperative recovery, and resumed surfing

EC-IC bypass, first described by Crowell and Yasargil, involves anastomosing an extracranial artery to a distal branch of the intracranial artery on which the IA is located [24]**.** The bypass allows safe ligation of the IA’s parent artery and hence subsequent obliteration of the IA for complex cases in which clipping is technically challenging [112]**.** There are two kinds of EC-IC bypass: low-flow and high-flow bypass. Low-flow bypass involves anastomosing the superficial temporal artery to an intracranial artery (STA-IC) such as the MCA, while the high-flow bypass connects the common carotid artery or the external carotid artery to an intracranial artery (CCA-IC or ECA-IC respectively) with the use of a conduit such as the great saphenous vein (GSV) or the radial artery (RA) [51, 115]**.** In general, low-flow bypass is the more popular choice, as a smaller and more gradual increase in inflow rate compared to high-flow bypass results in decreased risk of hyperperfusion injury [142]**.** However, in certain complex IA cases, high-flow bypass may be necessary as it encompasses the potential to supply a more substantial amount of blood flow to the brain [105]**.** Sekhar et al. demonstrated satisfactory outcomes of high-flow bypass with an RA conduit in a series of 17 patients with IAs deemed inoperable with conventional microsurgical clipping (Fig. 7) [101]**.**

Currently, the use of EC-IC bypass to treat IAs is reserved for giant or complex IAs. Giant IAs, which constitute 2–5% of all IAs [65, 114]**,** often need to be surgically removed to alleviate symptoms due to mass effect. EC-IC bypass allows continuation of regional perfusion in areas distal to the IA after removal. More recent literature has reported increasing use of bypass techniques with improved patient outcomes [114]**.** In the earlier series, 30-day surgical mortality was high, but remained low at 1% or less in the more contemporary surgical series [16, 45, 46, 48, 82, 103, 124]**.** In one of the largest bypass series that included 93 patients with giant IAs (46% involving ICA, 32% MCA, 18% posterior circulation), the bypass patency rate was 96%, and long-term excellent or good outcome was observed in 94% of patients, at a mean follow-up of 3 years [103]**.** As such, EC-IC bypass is a viable consideration in cases where the IA is not amenable to microsurgical clipping or endovascular coiling.

**Fig. 8:**
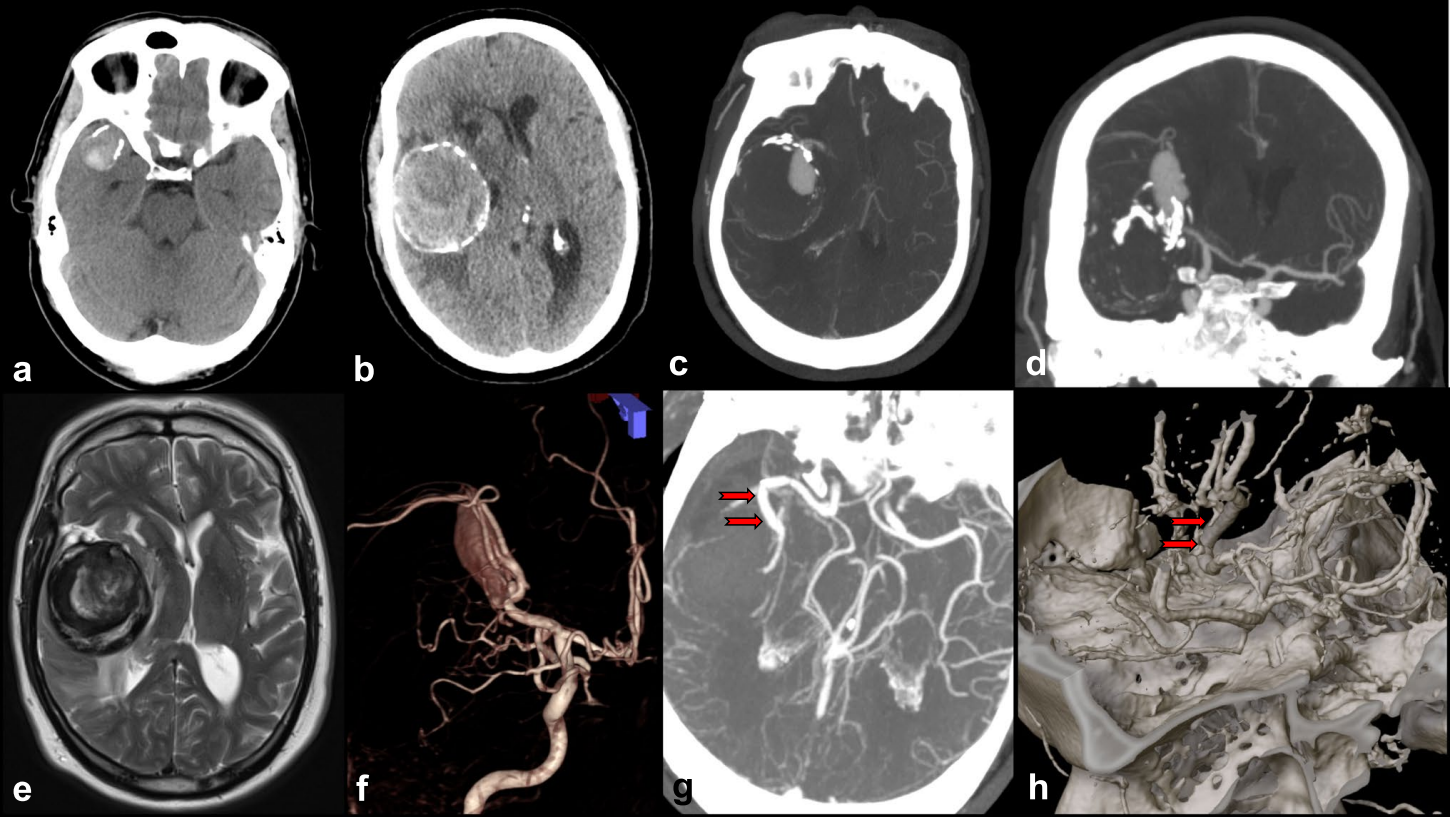
A 65-year-old man, hypertensive, smoker, and with previous TIAs, was diagnosed with a large right MCA aneurysm 13 years prior that was managed conservatively (**a**). He presented with progressive left hemiparesis, impaired conscious level. **b** CT head showed a heavily calcified giant right MCA aneurysm with surrounding edema. **c**, **d** CT angiogram (axial and coronal views) showed patent aneurysm remnant, heavy calcification at the aneurysm neck, and thick layers of intraaneurysmal thrombus. **e** MRI brain, T2-weighted, axial view showed onion-ring appearance of multilayered intraaneurysmal thrombus. **f** 3D reconstructed angiogram showed single MCA branch arising from the aneurysm neck. **g** He underwent aneurysmectomy with in situ MCA end-to-end anastomosis using interposition saphenous vein graft (IC-IC bypass) and made good recovery. At 6 months follow-up, he was independently mobile, with resolution of hemiparesis. **g**, **h** CT angiogram confirmed patent in situ bypass graft (red arrows)

**Fig. 9:**
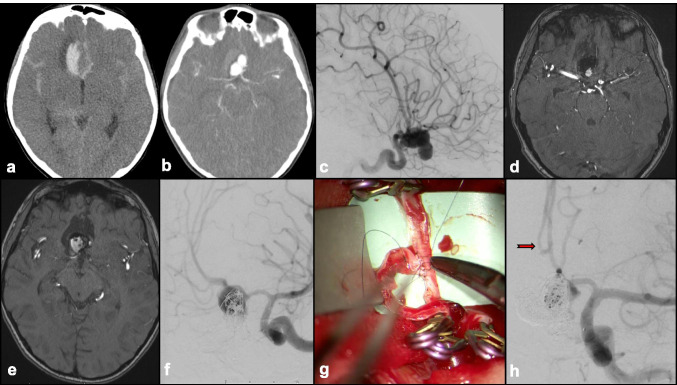
A 10-year-old patient presented with coma producing aneurysmal SAH. Preintubation, his best motor score was flexing. **a** CT head showed diffused SAH, with associated frontal hematoma. **b** CT angiogram confirmed an underlying complex multilobulated anterior communicating artery (ACommA) aneurysm. **c** Cerebral angiogram (R ICA injection) showed the multilobulated ACommA, and right A2 arising from the neck of the aneurysm. He underwent coil occlusion of the ruptured ACommA, securing the ruptured fundal component. **d** MR angiogram, small neck remnant of coiled aneurysm was evident to protect the right A2 branch. After a period of ICU stay and rehabilitation, he made good recovery. **e** MR angiogram at 6 months follow-up showed significant aneurysm recurrence. **f** Cerebral angiogram (left ICA) injection showed ACommA aneurysm enlargement. After multidisciplinary discussion, we proceeded to perform right pericallosal to left pericallosal in situ (IC-IC) bypass, prior to further coil occlusion of the aneurysm, sacrificing the right A2. **g** Intraoperative view of pericallosal-pericallosal in situ bypass (IC-IC), end to side technique using interrupted 9/0 suture. **h** Angiogram after further coil occlusion of the recurrent ACommA aneurysm, and left A2 supplying both pericallosal branches (red arrow)

The role of IC-IC bypass in IA surgery was introduced more recently (Figs. 8 and 9). IC-IC bypass involves revascularization and anastomosis of distal efferent branches with donor arteries that are completely in situ. Grafts such as the GSV or the RA may be used when a tension-free anastomosis is not possible, such as in the event that the donor and recipient vessels do not lie in close proximity [59]**.** While IC-IC bypass is a more technically challenging procedure, it is associated with higher IA obliteration rates, and higher bypass patency rates compared to EC-IC bypass according to some authors [99]**.**

## Hybrid techniques: combination of both microsurgery and endovascular therapies

**Fig. 10:**
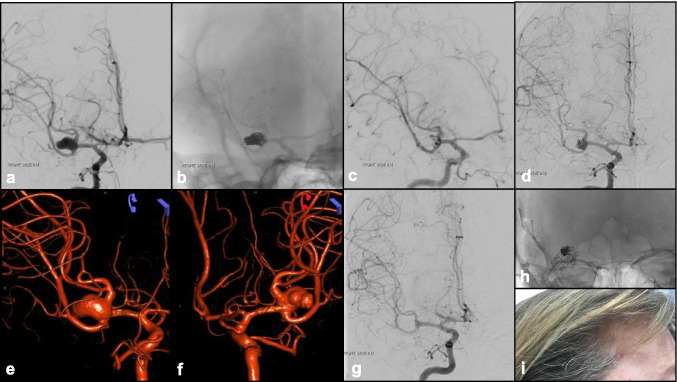
A 50-year-old patient with poor-grade SAH, treated by hybrid approach. **a** Cerebral angiogram (right ICA injection) confirmed a complex right MCA aneurysm. **b**, **c** Post-coiling angiogram showed satisfactory aneurysm occlusion, with protection of the fundal bleeding point. **d** Six months follow-up angiogram showed aneurysm neck recurrence/coil compaction. **e**, **f** 3-Dimensional reconstructed angiogram (AP, PA views) showed the complex wide-necked MCA aneurysm configuration with multiple daughter sacs. **g** Post-clipping angiogram confirmed complete right MCA aneurysm obliteration, using **h** clip reconstruction technique preserving MCA branches (**i**), via mini-pterional approach

Microsurgery and endovascular therapies are often viewed as competing treatments, but it is important to recognize their individual limitations. The ways in which endovascular techniques can be integrated into neurosurgical management, and vice versa, to enhance therapy have been underappreciated. As a result, controversy continues regarding the optimal management of IAs. A complex IA sometimes cannot be completely resolved with a single approach and its successful treatment requires a combination of microsurgical and endovascular techniques. Either technique could be a planned adjunct to the other or may be employed due to the primary failure of the other therapy. In this section, we will discuss how these two techniques could complement each other (Fig. 10).

### Microsurgical treatment post-endovascular therapy

In the setting of a ruptured IA, microsurgery can be challenging due to complications such as hydrocephalus, brain edema, altered cerebral hemodynamics, and rarely even a subdural hematoma necessitating immediate hematoma evacuation with insertion of external ventricular drain [119, 138]. In such cases, microsurgical clipping may be used as a more definitive therapy after initial coil embolization which is better tolerated in the acute setting. As sometimes ruptured IAs have unfavorable anatomy for cure with coiling, a common strategy in this setting is IA dome coiling for rupture protection immediately post-rupture with a planned future definitive curative procedure in a delayed manner. In this setting, adjunct microsurgical clipping may be used as a the definitive therapy if the patient recovers and has a known neck residual or there is IA regrowth on a delayed basis [128].

In the setting of severe vasospasm in SAH, it would be wise to avoid initial definitive microsurgical manipulation of vessels. Partial coiling followed by staged definitive clipping could be a better strategy to employ but access is often an issue if there is severe vasospasm [12].

Of course, this strategy is not amenable to all types of IAs. Partially coiled IAs can be structurally difficult to manipulate. If coils extend to the IA neck, they can hold the IA walls apart and prevent closure of the neck. This can potentially transform the soft neck into a wedge. If an adjunct clip were to be employed, it could lose traction and be splayed, resulting in it sliding down the neck and occluding parent and branch vessels. Thrombus formation within coils may also solidify the IA. In some cases, the IA dome may need to be opened with some coil and thrombus content removal before clipping can be safely performed [128].

Performing a preoperative endovascular balloon test occlusion (BTO) of a proximal parent vessel prior to microsurgical vessel sacrifice can be extremely beneficial in cases of complex IAs. BTO is usually only reserved for large vessels such as the ICA and vertebral artery, as it has a high risk or complication in smaller vessels. The apprehension of the risk of therapeutic artery sacrifice has urged preprocedural risk evaluations. Temporary BTO is a provocative test that transiently arrests blood supply to the areas that would be permanently affected following a vessel sacrifice. BTO is now widely used in patients with complex IAs as it helps determine which patients may require a bypass and whether a high- or low-flow bypass may be needed. A systematic review conducted by Linskey et al. showed that applying BTO prior to ICA occlusion considerably reduces the incidence of postoperative stroke and mortality from 26 and 12 to 13% and 3%, respectively [64]. In certain cases, BTO should be avoided as it might propagate dissection or thromboembolic complications.

### Endovascular therapy post-microsurgical treatment

A number of IAs are treated with clipping given their wide-based morphology which makes stable coil constructs difficult. Microsurgical treatment is invasive and is frequently avoided in high-risk and high WFNS grade aneurysmal SAH patients, owing to prolonged brain retraction, difficulty of vessel dissection, and the need for longer anesthesia. Compared to endovascular coiling, more brain manipulation and hemodynamic changes are implicated in microsurgical clipping. Postoperative cerebral blood flow and cerebral metabolic rate for oxygen may be decreased in the postoperative period secondary to brain retraction [137].

Microsurgical clipping allows protection of a large part of the IA, but in complex IAs, due to a limited surgical corridor, a segment of the IA neck may be hidden from view, resulting in residual IA filling after the IA neck is clipped [21]. In such settings, a partial microsurgical clip reconstruction of the IA can help create a narrow neck, which makes subsequent coil embolization of the residual IA possible. This scenario is encountered in carotid cave and paraclinoid IAs where part of the IA extends beyond the dural ring. This unique anatomy prevents placement of a microsurgical clip without dissection of the cavernous sinus, which can have significant morbidity. Reconstruction of a narrow neck permits easy coiling of the IA. While neck reconstruction to favor coiling can help, it can alter flow dynamics with a higher flow jet toward the IA dome, increasing likelihood of rupture. Therefore, second stage coiling should be completed soon after clip reconstruction. Wrapping with muslin may afford protection from rupture until subsequent coiling [20].

A common scenario for the use of coil embolization after clipping is with IA recurrences. Patients with an enlarging IA residual can be treated with coiling, possibly with balloon and stent assistance. This prevents the need for reoperation for a previously clipped IA and avoids issues with scarring and high surgical risk.

## To coil or to clip? What is the best available evidence?

The emergence of endovascular therapy has inevitably reduced cases of open surgery [73]. Endovascular treatment is preferred for certain posterior circulation IAs; however, surgery is a durable and effective option for certain IAs, particularly MCA IAs [140]. Although traditionally deemed as competitors, both microsurgical and endovascular approaches should complement each other.

The ISAT and Barrow Ruptured Aneurysm Trial (BRAT) were the only large randomized controlled trial (RCT) that compared clipping with coiling in patients with ruptured IAs, suitable for either treatment [73, 110]. However, results of the ISAT have continued to spark debates and controversy, mainly because of its selection bias. The vast majority of patients had a favorable grade at the time of enrolment, 95% of the IAs were in the anterior cerebral circulation, and 90% were < 10 mm, which made these results difficult for clinicians to generalize to treatments of specific IAs. As explained, there is no blanket technique to treat all kinds of IAs, due to variations in morphology, anatomy, location, and rupture status. The decision made would have to be guided by best available evidence, operator experience, and finally individualized joint decision between the patient and multidisciplinary teams composed of neurosurgeons, neurologists, critical care physicians, and interventional neuroradiologists. Since then, more RCTs, as well as observational studies, have been published, some of which have results that differ from that of the ISAT. Ten-year analysis from the BRAT demonstrated that, after 1 year, clinical outcomes for patients with anterior circulation IAs were comparable in both groups; clipping had several advantages over endovascular treatment, including greater rates of complete IA obliteration and reduced rates of retreatment [110]. Hence, more randomized and long-term data are needed.

The Cochrane review by Lindgren et al. on this topic only included four RCTs and the results principally favored those of the ISAT, due to its dominance in patient numbers [63]. There have been other published meta-analyses comparing the two treatment modalities, which include data from observational studies, specifically in the treatment of patients with unruptured MCA IAs [3], and patients with oculomotor nerve palsies induced by posterior communicating artery IAs [143]. We summarize in Table 3 the systematic reviews and meta-analysis which are most up-to-date or comprising largest dataset, comparing outcomes (efficacy, complications rates, morbidity, mortality, and economic status) of microsurgical and endovascular approaches for IAs by type, location, and rupture status.

**Table 3:** Summary of the most up-to-date (or comprising largest dataset) systematic reviews and meta-analyses comparing outcomes of microsurgical and endovascular approaches for IAs, stratified by rupture status, cost analysis, and aneurysm location

Author (year)	Patient demographics	Included study characteristics	Follow-up duration	Conclusions
All locations				
Li et al. (2013)	11,568 patients with ruptured IAs Mean age of patients 45 to 58	4 RCTs 23 cohort studies (7 prospective; 14 retrospective; 2 ambidirectional)	12 months	• Coiling yields better clinical outcomes than coiling, the benefit being greater in those with a good preoperative grade (WFNS grades 1–2, or Hunt & Hess scale 1–3)—poor outcome mRS 3–6/ GOS 1–3 rate of coil versus clip: 26.8% versus: 30.3% • However, incidence of rebleeding is higher after coiling (OR = 0.4) • The mortality of the two treatments shows no significant difference within 12 months (10.4% versus 8.5%)
Lindgren et al. (2018)	2458 patients with ruptured IAs	4 RCTs	12 months	• Coiling is associated with a better outcome (poor outcome rate mRS 3–6/ GOS 1–3 coil versus clip: RR = 0.77), for patients in good clinical condition with ruptured IAs of either the anterior or posterior circulation, suitable for both clipping or coiling
Delgado et al. (2017)	225,772 patients with ruptured (117,495) or unruptured (103,274) IAs	9 RCTs 76 observational studies	Short term < 3 months; intermediate > 3–12 months; long term > 12 months	• Coiling favored higher independent outcome (mRS 0–2/ GOS 4–5) (OR = 0.67, at short term; OR = 0.80 at intermediate follow-up; OR = 0.81 at long-term follow-up) • Coiling favored lower mortality at short term (OR = 1.74)
Hwang et al. (2012)	31,865 patients with unruptured IAs	24 observational studies	Short term, ≤ 6 months; long term, > 6 months	• Clipping is associated with significantly greater disability as seen on GOS in the short term (OR = 2.72), but not in the long term • Clipping is also associated with significantly greater rates of neurological and cardiac complications (ORs 1.94 and 2.51, respectively)
Kang et al. (2020)	129,317 patients with unruptured IAs	1 RCT 24 cohort studies (8 prospective; 147retrospective)	Short term, ≤ 30 days; long term, 12 months	• Surgical clipping results in lower retreatment rates (OR = 0.3) and is associated with a higher incidence of complete occlusion in both short-term (OR = 0.18) and 1-year follow-up (OR = 0.3) • Endovascular coiling is associated with shorter LOS (WMD = − 4.14) and a lower rate of short-term complications (OR = 0.52), especially ischemia
Anterior location only				
Jiang et al. (2020)	94,529 patients, 44,715 ruptured, and 49,814 unruptured intracranial aneurysms	7 RCTs 57 cohort studies (prospective; retrospective)	84 months	• Clipping is superior to endovascular coiling for ruptured IA in terms of mortality (OR = 0.8) and rebleeding (OR = 0.4) and complete occlusion rate (incomplete occlusion OR = 0.2) • Clipping is associated with higher incidence of poor outcome (OR = 1.4) and bleeding (OR = 1.7) compared with coiling for unruptured IA
Alreshidi et al. (2018)	1385 unruptured MCA aneurysm, 626 clipped, 759 coiled	37 case series		• Surgical clipping for unruptured MCA aneurysms remains highly safe and efficacious • Endovascular coiling for unruptured MCA aneurysms results in lower rates of complete occlusion (53% versus 94%).when compared with clipping
Zijlstra et al. (2016)	4300 MCA aneurysms (1891 ruptured and 2409 unruptured) in 4065 patients 2222 clipped, 2078 coiled	1 RCT 50 cohort studies (10 prospective; 40 retrospective)	Up to 108 months	• Both coiling and clipping are associated with low mortality and morbidity rates • Coiling may be better for ruptured aneurysms in terms of death rates (15% vs 8%) • Clipping may be better for unruptured aneurysms in terms of death rates (0.3% vs 1%)
Petr et al. (2017)	1329 DACA aneurysms (1050 ruptured and 223 unruptured; 56 were not classified) 786 clipped, 543 coiled	12 cohort studies 18 case series	NA	• Surgical treatment is associated with superior complete occlusion rates (95% vs 68%), compared with endovascular treatment • Surgical treatment is associated with lower rates of aneurysm recurrence (3% vs 19%), compared with endovascular treatment • There is no substantial differences in procedure-related morbidity, long term favorable neurologic outcomes and mortality between the two groups
Silva et al. (2017)	2458 patients with ruptured or unruptured paraclinoid aneurysms	39 cohort studies	26 months (clipped) 17 months (coiled)	• High rate of visual improvement without a significant difference in the rate of worsened vision or iatrogenic visual impairment with use of FD (71%) compared with clipping (58%) and coiling (49%)
Posterior location only				
Zheng et al. (2017)	297 patients with ruptured PCommA aneurysms	9 cohort studies	NA	• Clipping is superior over coiling for the complete recovery of ONP in patients with ruptured aneurysm of the PCommA (RR = 1.7)
Gaberel et al. (2016)	384 patients with ruptured or unruptured PCommA aneurysms Mean size 7.7 mm (clipped group), 7.4 mm (coiled group)	11 cohort studies	31 months (clipped) 39 months (coiled)	• Surgical clipping of PCommA aneurysms achieves better ONP recovery than endovascular coiling, especially for ruptured aneurysms (84% vs 43%)
Cost analysis^a^				
Zhang et al. (2018a)	49,181 ruptured IA (24,219 clipped, 24,962 coiled)	8 cohort studies (all retrospective) Data sources: Medicare Provider and Analysis Review; National Inpatient Sample; Health Insurance Review & Assessment Service; Premier Perspective Comparative Database; ISAT	12 months	• In the USA, total hospital costs (SMD = − 0.05) and 1-year medical costs (SMD = 0.15) are similar when treated with coiling or clipping • However, in a subgroup analysis revealed in South Korea and China, coiling is more expensive • The LOS is much shorter in coiled patients in all countries (SMD: 0.29)
Zhang et al. (2018b)	56,165 unruptured IA (24,856 clipped, 31,309 coiled)	9 cohort studies (all retrospective) Data sources: MarketScan; Health Insurance Review & Assessment Service; Health Share	12 months	• No significant difference in total hospital costs (SMD = − 0.33) and 1-year medical costs (SMD = − 0.04) between coiling versus clipping in both the USA and South Korea • However, the LOS after coiling is much shorter than neurosurgical clipping (SMD = 0.77)

*DACA*, distal anterior cerebral aneurysm; *FD*, flow diverters; *GOS*, Glasgow Outcome Scale; *IA*, intracranial aneurysm; *ISAT*, International Subarachnoid Aneurysm Trial; *LOS*, length of stay; *MCA*, middle cerebral artery; *mRS*, modified Rankin Scale; *NA*, not available; *ONP*, oculomotor nerve palsy; *PCommA*, posterior communicating artery; *RR*, risk ratio; *WFNS*, World Federation of Neurosurgical Societies

^a^Data from low-to-middle-income countries were not included; hence, the conclusions drawn would not be applicable to these countries

When making a decision of which treatment, the following factors are crucial to consider: (1) age, past medical history, and comorbidities help to determine the patient’s ability to tolerate a specific treatment. A young patient with a ruptured IA may be a good candidate for open surgery with better prospects of a long-term durable result, with lower rates of rebleeding [60]. In contrast, in an elderly patient with comorbidities with a ruptured IA and a diffusely swollen and friable brain, coil embolization may be favored over surgery. (2) IA characteristics such as location (suitability for surgical access), morphology, and size. Basilar tip IAs are notorious to treat with open surgery and are associated with higher surgical morbidity compared to IAs of other locations, so endovascular treatment is usually the preferred choice of treatment, although many indications still exist for the use of microsurgery [123]. MCA IAs typically have wide necks with important perforating branches originating from its dome or base. In such cases, endovascular coiling may not be performed safely without compromising flow. In such a scenario, surgery offers the option of clip reconstruction of the IA while preserving important perforators and bifurcation vessels. A ruptured IA with intraparenchymal hematoma causing significant mass effect is likely better suited for surgery. (3) Operator’s experience and preferences. This includes the operator’s bias toward a certain treatment because of experience and familiarity. (4) Finally, an equally important but often understated factor is the patient’s preference. Patients may prefer the cosmetic benefit of the minimally invasive endovascular approach but should always be informed of the short- and long-term risks associated with it. (5) It is also noteworthy that, on a global level, the higher direct cost of endovascular treatment is by far a major determinant of treatment selection in low- and middle-income countries (LMICs) [32]. In LMICs, this is of even more concern given the lack of access to much of the world’s population to surgical and, more specifically, neurosurgical, interventional, and anesthesiologic expertise [29, 80, 98].

## Evolution of intracranial aneurysm treatment techniques

The treatment of IAs has undergone very significant paradigm shifts in the recent decades. The introduction of any new device is inevitably accompanied by evolution of the technique as clinical experience grows and case selection improves. This phenomenon will continue to prevail with further technological advancements. This conversely can lead to the paradox of choice. With a large armamentarium of IA treatment techniques, one of the challenges of the operator may be to face achieving mastery in the surgical techniques or devices of their trade while keeping pace with the explosive evolution of technology and techniques. In addition, using a range of techniques may reduce their expertise in each individual technique. Any new technique assessed must account for the options that are available to the patient and the likely outcomes of those options. The technology should be generalized for use by most of the expert community, because, if it is too esoteric or technically demanding, it may not be commercially and clinically successful. Thus, the evaluation of new techniques should be carried out in multiple centers with a diversity of clinicians as this provides a more representative reflection of what can be expected when a device is made readily available. Future directions and innovation for IA treatment techniques will be based on the refinement of currently available technology and driven by what is identified as current limitations of established techniques. The ultimate aim of the evolution is to address current shortfalls and reduce complications and morbidity. For example, the advent of flow diverters has helped overcome prior limitations of SAC in the treatment of wide-necked, giant, or fusiform IAs. Understanding whether the new devices and surgical techniques are at least equally safe and potentially more effective than currently available treatments and whether IAs of different locations and subtypes can be treated safely and completely remains an ongoing challenge in cerebrovascular neurosurgery. Finally, addressing the cost issue of emerging technologies is also of major importance to enable application in LMICs [29, 80, 98].

## Conclusion

We summarized the available treatment options for IAs. The treatment should be approached in a multidisciplinary manner, taking into account the characteristics of the IA, patient’s comorbidities, the patient’s preferences, and the experience of the operator. The patient’s condition at presentation plays an important role in the decision-making by experienced neurovascular surgeons and endovascular specialists. In certain complex IA cases, a single approach may be inadequate to completely treat it, and its successful treatment requires a combination of microsurgical and endovascular techniques. Ultimately, it is intended that this review will allow the reader to apply the best matched evidence to decide the best approach towards a particular lesion.

## Data Availability

Not applicable.
